# The Effects of Pleiotrophin in Proliferative Diabetic Retinopathy

**DOI:** 10.1371/journal.pone.0115523

**Published:** 2015-01-24

**Authors:** Xuemei Zhu, Yujing Bai, Wenzhen Yu, Chungting Pan, Enzhong Jin, Dan Song, Qiong Xu, Yuou Yao, Lvzhen Huang, Yong Tao, Xiaoxin Li, Mingwei Zhao

**Affiliations:** Department of Ophthalmology, Peking University People’s Hospital, Key Laboratory of Vision Loss and Restoration, Ministry of Education, Beijing Key Laboratory of Diagnosis and Therapy of Retinal and Choroid Diseases, Beijing, 100044, China; Saitama Medical University, JAPAN

## Abstract

Pleiotrophin (PTN), a secreted, multifunctional cytokine, is involved in angiogenic, fibrotic and neurodegenerative diseases. However, little is known about its effects on diabetic retinopathy, a neurovascular disease. To investigate the role of PTN in proliferative diabetic retinopathy (PDR), PTN concentration in the vitreous was evaluated in PDR patients and non-diabetic controls. PTN expression was observed in epiretinal membranes from patients. PTN knockdown was performed using small interfering (si)RNA, and the effects on retinal pigment epithelium (RPE) cells and human umbilical vascular endothelia cells (HUVECs) were observed *in vitro* under hyperglycemic and hypoxic conditions. Cell attachment, proliferation, migration, tube formation, cell cycle, apoptosis, extracellular signal-regulated kinase 1/2 (ERK 1/2) phosphorylation, and VEGF levels were studied. The vitreous PTN concentration in PDR patients was higher than that in non-diabetic controls, and PTN was highly expressed in the fibrovascular membranes of PDR patients. Under hyperglycemic and hypoxic conditions, PTN knockdown reduced cell attachment, proliferation, migration, and tube formation and induced cell cycle arrest and apoptosis *in vitro*. Mechanically, PTN depletion decreased ERK 1/2 phosphorylation. Recombinant PTN up regulated the concentration of VEGF *in vitro*, which can be attenuated by the ERK 1/2 inhibitor. Taken together, our results implied that elevated PTN in PDR patients might participate in the critical processes of the development of PDR, most likely playing roles in angiogenesis and proliferation, possibly by activating the ERK 1/2 pathway and regulating VEGF secretion. These findings provide new insight into the roles of PTN in PDR and suggest that PTN may become a new target for therapeutic intervention in PDR.

## Introduction

Diabetic retinopathy (DR) has become one of the leading causes of visual impairment and blindness worldwide, and one-third of diabetic patients suffer from this complication[1,2,3]. Although several pathways have been proposed, the precise pathogenesis of DR is not completely known. Neovascularization is the predominant feature of proliferative diabetic retinopathy (PDR), and hyperglycemia is considered to be the key factor responsible for the development of diabetic vascular complications. Studies have indicated that cell dysfunction in the retina is induced by hyperglycemia through increases in the levels of many types of growth and inflammatory factors. Notable among these cells are endothelial and retinal pigment epithelium (RPE) cells, which form part of the blood-retinal barrier and perform maintenance functions in the visual system[4,5,6,7]. Cell injury leads to retinal ischemia and hypoxia and further aggravates the progression of DR. Moreover, recent studies have shown that loss of photoreceptors is an early change in DR and that neuronal cell apoptosis[8] and glial dysfunction[9] lead to various vascular changes[10,11,12]. Several studies have shown that neuroprotective agents can delay the progression of DR[13,14]. These findings suggest that DR may be a neurovascular disorder and not just a vascular disease.

Until recently, limited methods, including photocoagulation, anti-VEGF, and triamcinolone acetonide, have demonstrated efficacy in the treatment of DR[15,16,17,18]. However, some cases have shown no response to these regimes[19]. These observations indicate that there may be other mechanisms involved in the development of DR. Considering that DR is a neurovascular disease, devising novel strategies targeting both angiogenesis and neuroprotection is therefore important, as such treatments may delay or even stop the formation and progression of DR at a very early stage.

Pleiotrophin (PTN), an effective angiogenic and neurotrophic factor, is overexpressed in many pathologic conditions. PTN is an 18-kDa secreted growth factor, also called heparin affin regulatory peptide (HARP) or heparin-binding growth-associated molecule (HB-GAM), that binds five cell surface receptors: protein tyrosine phosphatase receptor (RPTPβ/ζ), anaplastic lymphoma kinase (ALK), syndecan-1, syndecan-3, and syndecan-4[20]. Together with midkine, PTN forms a family of heparin-binding proteins that are normally expressed temporally and spatially[21]. PTN has been widely identified in various human tumors exhibiting a proto-oncogene function. Not only is PTN involved in tumorigenesis by enhancing the angiogenesis and proliferation of the tumor cells[22,23,24], but it also affects perineural invasion and metastasis by remodeling the malignant cell microenvironment[25,26,27]. Different approaches that abrogate constitutive PTN signaling in the malignant cells effectively reverse tumor angiogenesis[22,28]. In addition, PTN has also been investigated in fibrotic tissues[29], hypertrophic scars[30] and rheumatoid arthritis[31], where it is involved in fibrogenesis and inflammation[32]. Moreover, as an effective neuroprotective factor, PTN is highly up-regulated in neurodegenerative disorders[33]. Studies have demonstrated that PTN stimulates neurite outgrowth from cultured neurons [34], induces a bipolar form of glial cell precursors[35], promotes axonal growth and regeneration, and supports neuronal survival[36,37]. These findings suggest that PTN may play a vital role in DR which is considered to be a neurovascular disease. However, whether PTN plays a role in DR has not yet been explored.

In the present study, we characterized PTN expression in the vitreous fluid and proliferative membranes of PDR patients and explored its possible roles under hyperglycemic and hypoxic conditions to simulate the *milieu interne* of PDR patients *in vitro*. Our results demonstrated that elevated PTN in PDR likely plays a role in angiogenesis and fibro-proliferation by activating the ERK 1/2 pathway and regulating VEGF secretion. Thus, PTN may become a new target for therapeutic intervention in PDR in the future.

## Methods

### Subjects and sample collection

The Ethics Committee and Institutional Review Board of Peking University (Beijing, China) approved this human study protocol, and written informed consent was obtained from each study subject in accordance with the Declaration of Helsinki. All of the subjects received a standard ophthalmic examination by two retinal specialists (Dr. Wenzhen Yu and Dr. Mingwei Zhao). Twenty-nine patients with PDR who underwent pars plana vitrectomy were enrolled in the study, and 5 of them received anti-VEGF treatment 14.8±11.1 days before surgery. The controls were 14 eyes of non-diabetic patients (idiopathic macular hole [IMH]; III/IV stage, Gass IMH phases, n = 9; and idiopathic epiretinal macular membrane [EMM], n = 5). Vitreous samples and epiretinal membranes were surgically removed from patients when undergoing pars plana vitrectomy. Membrane tissues were placed in phosphate-buffered saline (PBS; pH 7.4), mounted in optimal cutting temperature medium (Merck, Darmstadt, Germany), and 6-μm sections were cut. Undiluted vitreous samples (0.3 ml to 0.5 ml) were obtained at the onset of vitrectomy by aspirating through the vitreous cutter under the simultaneous inflation of the vitreous cavity with air through the infusion cannula. The vitreous samples were transferred to a sterile tube, placed immediately on ice, and centrifuged at 15,000 g for 5 min at 4°C to remove cells and debris. Supernatants were frozen at −80°C until the assay was performed. Dr. Wenzhen Yu, Dr. Yong Tao, and Dr. Mingwei Zhao at Peking University People’s Hospital performed all of the surgeries.

### Immunofluorescence staining of membranes

Tissue sections were fixed in 4% paraformaldehyde (PFA). After blockading, anti-PTN polyclonal antibodies (dilution 1:50, Cat#ab95391, Abcam, Cambridge, UK) with anti-CD31 antibodies (dilution 1:100, cat#ab9485, Abcam) were applied to the tissue sections at 4°C overnight and incubated with tetramethylrhodamine (TRITC)-conjugated goat anti-mouse and fluorescein isothiocyanate (FITC)-conjugated goat anti-rabbit secondary antibodies (ZSGB-BIO, Beijing, China). After incubation, the slides were washed, and the cell nuclei were stained with 4, 6-diamino-2-phenylindole (DAPI, Roche Diagnostics, IN). Images were acquired with a confocal laser-scanning microscope (LSM710; Zeiss, Oberkochen, Germany). For each of the immunostaining procedures, negative controls included omission of the primary antibody and use of an irrelevant polyclonal or isotype-matched monoclonal primary antibody; in all cases, negative controls showed only faint, insignificant staining.

### Measurement of PTN and VEGF concentrations by ELISA

The PTN concentration in the vitreous body was measured with an enzyme-linked immunosorbent assay (ELISA) using a human PTN ELISA kit (Cat#E91309Hu, Uscn, China) in accordance with manufacturer’s instructions. The VEGF concentrations in the vitreous and cell supernatant were also measured with an ELISA using a human VEGF_165_ ELISA kit (Cat#EK0575, Bostar, Wuhan, China). Each assay was performed according to the manufacturer’s instructions. PTN and VEGF concentrations were expressed as pg/mL.

### Cell culture and transfection assays

HUVECs and human RPE cells (ARPE 19) (American Type Culture Collection [ATCC], Manassas, VA) were used in this study, as previously reported[38,39]. PTN (GenBank accession no. NM002825)-specific small interfering(si)RNAs (PTN-siRNA) were purchased from Santa Cruz Biotechnology, Inc. (Cat#sc-39713, California, USA). VEGF (GenBank accession no. NM003376)-specific siRNAs (VEGF-siRNA: forward: 5′-GGCAGAAUCAUCACGAAGUTT-3′; reverse: 5′-ACUUCGUGAUGAUUCUGCCTT-3′) were chemically synthesized by GenPharma (Shanghai, China). RPE cells and HUVECs were transfected with siRNA (Hiperfect Transfection Reagent; Qiagen, Hilden, Germany) according to the manufacturer’s instructions. Briefly, on the day of transfection, cells were seeded in plates at the recommended densities with 33 mmol/L glucose (Beijing Chemical Works, China) and hypoxia (5% O_2_). siRNA was then gently introduced into the cells by mixing with the required amount of transfection reagent at a final concentration of 10 nM siRNA. Non-silencing siRNA (NS-siRNA) (Cat#301799, Hiperfect, Qiagen) was used to control for any effects of the transfection reagent and siRNA. The *in vitro* assays described herein were performed 48 h after transfection under hyperglycemic and hypoxic culture conditions, including assessment of cell attachment, proliferation, migration, tube formation, cell cycle, apoptosis, ERK 1/2 phosphorylation, and PTN mRNA levels. All other reagents were purchased from Sigma-Aldrich (St. Louis, MO, USA).

### RNA isolation and real-time PCR

Total RNA was isolated (Trizol; Invitrogen, Carlsbad, CA) according to the manufacturer’s instructions. Reverse transcriptase reactions were performed using the RevertAid First Strand cDNA Synthesis Kit with oligo-dT primer (Fermentas, Pittsburgh, PA). Real-time PCRs were performed with SYBR Green PCR mix (Thermo, Pittsburgh, PA) using an ABI7300 real-time PCR system (Applied Biosystems, Life Technologies, Foster City, CA). The primers used in real-time PCR were PTN: forward 5′-CCAACTCAAAAATGCAGGCTCA-3′; and reverse 5′-CCACTGCCATTCTCCACAGT-3′; VEGF: forward 5′-GTTCAGAGCGGAGAAAGCA-3′; and reverse 5′-TCACATCTGCAAGTACGTTCG-3′; and glyceraldehyde 3-phosphate dehydrogenase (GAPDH): forward 5′-GAGTCCACTGGCGTCTTCAC-3′; and reverse 5′-GTTCACACCCATGACGAACA-3′. For each primer set, variability was assessed in 3 to 5 independent PCR runs; PTN was normalized to GAPDH expression and calculated using the equation: change (x-fold) = 2^−ΔΔCt^.

### Immunocytochemistry assay

RPE cells and HUVECs grown on glass coverslips were washed and fixed with 4% PFA in PBS and then permeabilized with 0.1% Triton X-100 before blocking with 10% goat serum. The slides were incubated with anti-PTN antibodies (dilution 1:80, Cat#ab95391, Abcam) at 4°C overnight and were then incubated with anti-rabbit FITC-conjugated secondary antibodies. Cell nuclei were stained with DAPI. Images were acquired with a fluorescence microscope (Zeiss Axiophot, Thornwood, NY). In each case, pre-immune IgG and secondary control incubations were conducted to determine the staining specificity. In each case, pre-immune IgG and secondary control incubations were conducted to determine the staining specificity.

### Cell attachment and cell proliferation

Cell lines were plated at 1×10^4^ per well in 96-well plates, and transfection was performed as described above. Forty-eight hours later, cell proliferation was measured with a Cell Counting Kit-8 (CCK-8, Dojindo, Tokyo, Japan) assay in accordance with the manufacturer’s instructions and as previously reported[38]. For the cell attachment assay, 96-well plates were coated with 1.2 mg/ml of fibronectin in 100 ml PBS overnight at 4°C. The plates were blocked with 2.5 mg/ml BSA for 2 h in DMEM or RMPI-1640 at 37°C. Transfected cells were trypsinized, and 1.5×10^4^ cells were seeded in each well for 1 h at 37°C. Cells were then washed two times with PBS, and the unattached cells were discarded. After the washing step, the number of attached cells was determined by a CCK-8 assay as described previously. Each experiment was repeated in five wells and was duplicated at least three times.

### Migration assay

Cell migration was analyzed using Transwell plates (Cat#3422; Corning, Tewksbury, MA), as described previously[38]. Briefly, 2×10^5^ cells were placed in the top part of a Transwell plate with serum-free medium after transfection. Medium containing 10% FBS was placed in the bottom chamber. All migration assays were conducted at 37°C for 6 h, and the cells were then fixed with 4% PFA and stained with DAPI. The cells that had not migrated were removed with a cotton swab, and the membrane was imaged with fluorescence microscopy. Cells from five random view fields were counted, and the average was used for statistical analysis.

### Tube formation study

According to the manufacturer’s instructions and our previous report[38,39], 150 μl of Matrigel (Cat#354234, BD Sciences, Franklin Lakes, NJ) solution was poured into 48-well plates and was then incubated at 37°C for 30 min. The pretreatment HUVECs (5×10^4^ per well) were seeded on the Matrigel and cultured for 4–8 h. The networks in the Matrigel from five randomly chosen fields were counted and photographed. The length of the tube was measured using ImageJ software (National Institutes of Health, Bethesda, MD). The experiments were repeated three times.

### Flow cytometry analysis of cell apoptosis and the cell cycle

A cell apoptosis study (FITC Annexin V Apoptosis Detection Kit; BD Science) and cell cycle analysis (Cycletest Plus DNA Reagent Kit; BD Science) were performed according to the manufacturer’s instructions and as previously reported[38]. Briefly, RPE cells and HUVECs (1×10^6^) were seeded in six-well plates and transfected with NS-siRNA and PTN-siRNA for 48 h under hyperglycemic and hypoxic conditions. The samples were analyzed using flow cytometry (FACSCalibur; BD Biosciences); the experiments were performed in triplicate and repeated three times. The apoptotic rate was calculated as the percentage of early apoptotic cells (LR) plus late apoptotic cells (UR).

### Western blot analysis

RPE cells and HUVECs were treated as described above. The cells were then prepared using protein extraction and protease inhibitor kits (Pierce, Rockford, IL), and lysates were cleared by centrifugation at 12,000 g at 4°C. The supernatants were collected, and the protein content of each lysate was measured with a BCA protein assay kit (Pierce) according to the manufacturer’s instructions. Equal amounts of protein were loaded and analyzed by immunoblot. The proteins were visualized with enhanced chemiluminescence Western blot detection reagents (Pierce). Phosphorylated-p44/42 mitogen-activated protein kinase was detected using a primary antibody (phospho-p44/42 MAPK [p-ERK 1/2], 1:2000; Cat#4370, Cell Signaling Technology, Danvers, MA) and an HRP-conjugated secondary antibody (goat anti-rabbit IgG; 1:6000; ZSGB-BIO, Beijing, China); band densities were quantified by densitometry using Image J software (National Institutes of Health, Bethesda, MD) and were normalized to GAPDH. Western blot analyses were repeated three times, and qualitatively similar results were obtained.

### VEGF_165_ measurement by ELISA in RPE cells and HUVECs stimulated by PTN

VEGF is the most important growth factor in the pathophysiology of PDR. To evaluate the relationship between PTN and VEGF, an ELISA was used to detect the concentration (pg/ml) of VEGF in the cell culture supernatant. RPE cells and HUVECs were incubated in fresh serum-free medium for 24 h before use in experiments. Recombinant PTN (rPTN) (Cat#RPB309Hu01, Uscn, Wuhan, China) was incubated with cells in 24-well plates for 1, 4, 6 and 24 h at concentrations of 0, 10, 50, 100, and 200 ng/ml[40] in general culture medium (10% FBS). In the other group, cells were treated with the pharmacological inhibitor U0126 (10 μM; Cat#S1102, Selleck Chemicals, USA) half an hour before the same rPTN treatment process. After the incubations, the cell culture supernatant was harvested and centrifuged. Secreted VEGF_165_ protein in the culture medium was measured with an ELISA according to the manufacturer’s instructions as described above. All of the experiments were performed in 5 pups, and each experiment was repeated three times.

### Statistical analysis

The data analysis was performed using Prism 6 statistical software (GraphPad Software Inc., San Diego, CA) and SPSS (SPSS, version 16.0; SPSS Science, Chicago, IL). All of the data are presented as the means±SD, and the normality of the distribution was assessed. The differences were evaluated with an analysis of variance (ANOVA), followed by the Student-Newman-Keuls test for multiple comparisons and Student’s t-test for pair-wise comparisons. The difference in intravitreous PTN concentrations in the two groups was analyzed by univariate analysis of covariance with age as the covariate. A P <0.05 was considered to be a statistically significant difference.

## Results

### Detection of PTN in proliferative membranes from PDR patients

**Figure 1:**
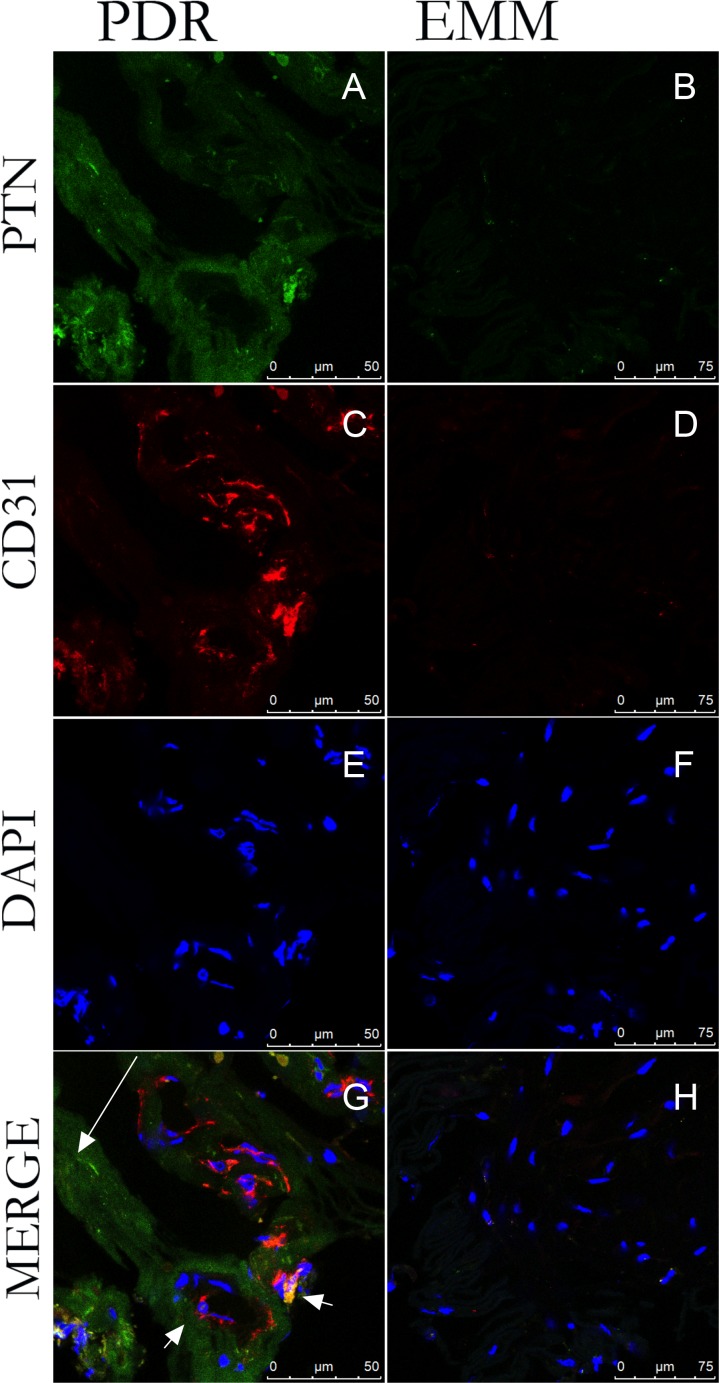
Detection of PTN on epiretinal membranes from PDR patients. Micrographs show immunofluorescence double staining of epiretinal PDR membrane sections (left column) and EMM sections (right column) for PTN (A, B) and CD31 (C, D). Nuclei were stained with DAPI (E, F). Images in the fourth row are merged (G, H). Double staining revealed PDR membranes expressing PTN and CD31. PTN expression was detected in CD31-labeled cells (panel G, short arrow) and in fibrous-like tissue (panel G, long arrow). However, neither PTN nor CD31 was detected in EMMs. Bar, 50 μm.

The expression of PTN in proliferative membranes from 11 PDR patients and 5 EMM patients were observed by immunofluorescence. Immunofluorescence analysis revealed that PTN was expressed in all the proliferative membranes from PDR patients and was only slightly detectable in the EMMs. As shown in **Fig. 1**, PTN and CD31 were co-localized in the fibrovascular membranes from PDR. As CD31 is a biomarker of endothelial cells, the results revealed that PTN was expressed in the vessels. PTN was also detected in the fibrous-like tissues (**Fig. 1G**). However, we did not detect visible staining of PTN and CD31 in the EMMs. We have also stained PTN with glial fibrillary acidic protein (GFAP) in membranes from PDR, as shown in supplemental figure (**S1 Fig.**). The result revealed that PTN was detected around glia cells.

### Increased PTN levels in vitreous samples from PDR patients

**Figure 2:**
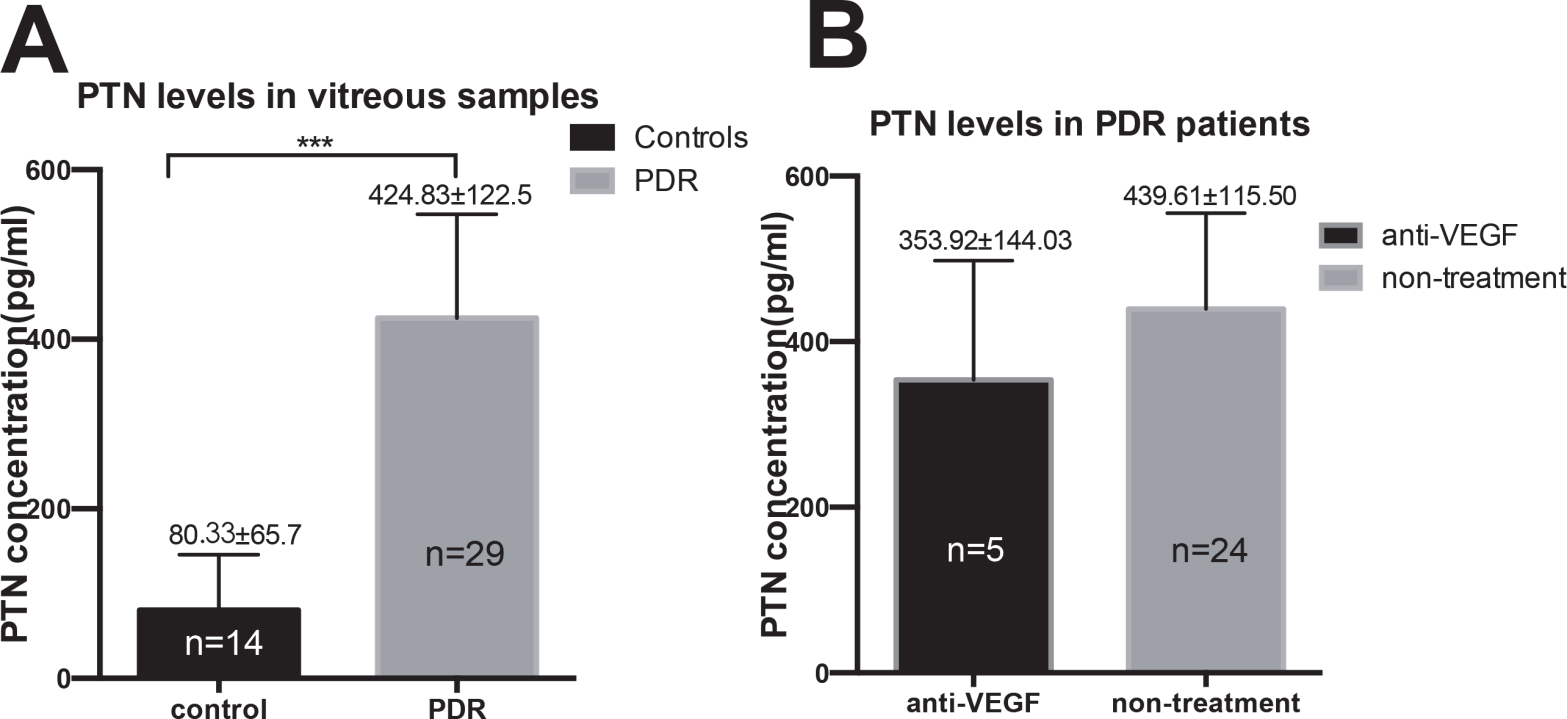
Concentration of PTN in the vitreous body from patients. Panel A shows the statistical results of PTN levels in vitreous samples from patients with non-diabetic retinopathy (idiopathic macular hole or idiopathic EMM) and from patients with PDR. PTN expression is significantly higher in the PDR group than that in the non-diabetic control group. Panel B shows the comparison results of the intravitreous PTN concentrations from patients with PDR who underwent anti-VEGF therapy or non-treatment before vitrectomy. The PTN concentration was not affected by the anti-VEGF treatment. Data are presented as the means ± SD (***P < 0.001).

The levels of PTN in the vitreous fluid were evaluated in 29 patients with PDR and 14 non-diabetic controls. PTN was detected in all vitreous samples from PDR patients and in 10 of 14 samples from non-diabetic control patients. The demographic, clinical, and laboratory data of subjects included in the study are illustrated in **Table 1**. The mean age of the PDR group was younger than that of the non-diabetic control group (P < 0.01). There was no difference in gender between PDR patients and non-diabetic control patients. The mean PTN level in the vitreous samples from the PDR patients was significantly higher than the mean level in non-diabetic control patients (P < 0.001; **Fig. 2A**).

**Table 1:** Demographic, clinical, and laboratory data of subjects included in the study.

**Variable**	**PDR group(n = 29)**	**Control group(n = 14)**	**P-value**
Age (years)	57.6±9.2	66.64±6.91	0.002^a^
Gender (male/female)	13/16	3/11	0.142^b^
Duration of diabetes (years)	11.57 (3—30)	—	—
Fasting blood-glucose	7.58 (3.13—11.82)	—	—
Intravitreous PTN (pg/ml)	424.83±122.5	80.33±65.7	< 0.001^c^

PDR: proliferative diabetic retinopathy, PTN: pleiotrophin. Data are expressed as the mean ± standard deviation or the median and range.

a. The P value was obtained by sample t-test.

b. The P value was obtained by chi-square test.

c. The P value was analyzed using univariate analysis of covariance with age as covariate.

Within the PDR group, 5 patients underwent anti-VEGF treatment before surgery. Although the VEGF level of these patients was very low, the PTN concentration was still high, and there were no significant differences in the PTN levels compared with other PDR patients who did not receive anti-VEGF therapy before surgery (P > 0.05, **Fig. 2B**).

### siRNA knocked down the expression of PTN

**Figure 3:**
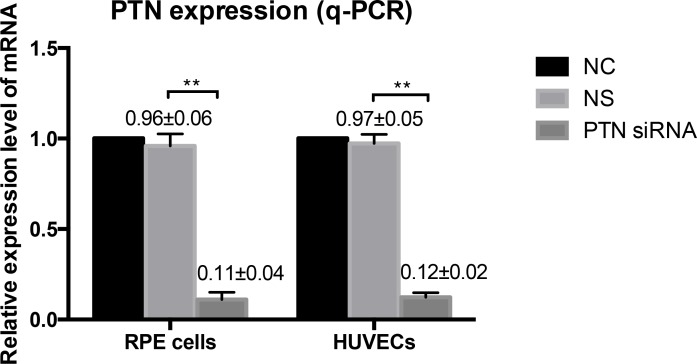
Effect of PTN-siRNA on RPE cells and HUVECs. PTN expression in human RPE cells and HUVECs was significantly knocked down in PTN-siRNA treated groups at the mRNA level, as measured by real-time RT-PCR 48 h after transfection. There’s no difference in NC and NS groups. The expression of NC was set to 100%. Data are expressed as the means ± SD of results from at least three independent experiments (**P < 0.01).

**Figure 4:**
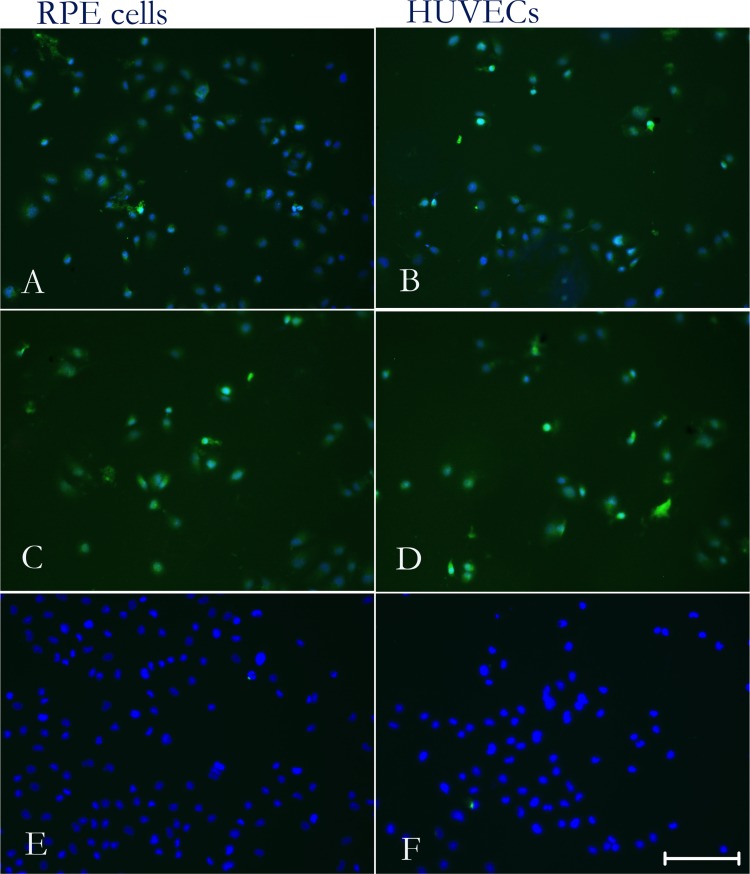
Immunocytochemical assays for PTN in RPE cells and HUVECs. The fluorescence (green), representing PTN expression in cells, was very strong in NC (A, B) and NS (C, D) cells but was barely detectable in the PTN-siRNA–treated cells (E, F), further confirming the effectiveness of PTN-siRNA transfection. Bar, 100 μm.

The effectiveness of PTN-siRNA transfection into RPE cells and HUVECs was determined by real-time RT-PCR and cell immunofluorescence. RT-PCR demonstrated that PTN-siRNA specifically reduced PTN mRNA levels in RPE cells and HUVECs (P < 0.01; **Fig. 3**). There was no significant difference between the non-silencing-siRNA-treated group (NS) and normal control (NC) cells (P > 0.05). Immunocytochemical imaging of PTN expression in cells further confirmed PTN knockdown (**Fig. 4**). The fluorescence intensity representing PTN expression in NC (**Fig. 4A, 4B**) and NS (**Fig. 4C, 4D**) was very strong, but the expression level in PTN-siRNA-transfected cells (**Fig. 4E, 4F**) was barely detectable. These results demonstrated that we successfully generated specific knockdown reagents that selectively target PTN in RPE cells and HUVECs.

### Cell attachment, proliferation, migration, and tube formation were inhibited by PTN deletion

**Figure 5:**
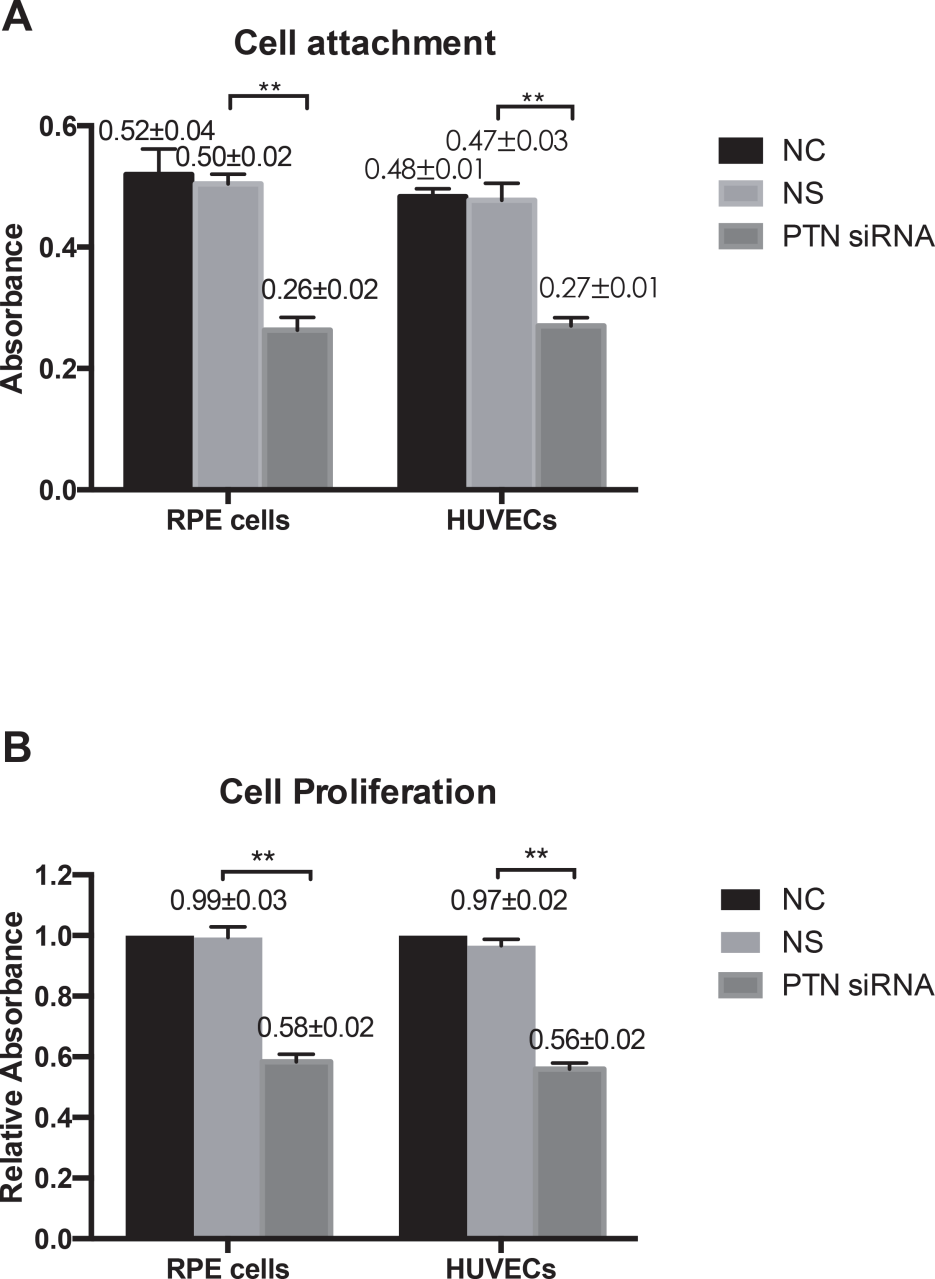
Effect of PTN on cell attachment and proliferation. Cell attachment was assessed after a 1-h incubation and subsequent CCK-8 testing. PTN-siRNA-treated cells reduced the adhesive capacities of RPE cells and HUVECs compared with the NS-siRNA-treated group. Cell proliferation was measured with a CCK-8 assay 48 h after transfection. PTN depletion significantly inhibited cell proliferation compared with the NC and NS groups. However, cell attachment and proliferation in the NC and NS groups were not significantly different. Data are the means ± SD of results from at least three independent experiments. **P < 0.01 vs. the control. NC was set to 100%.

**Figure 6:**
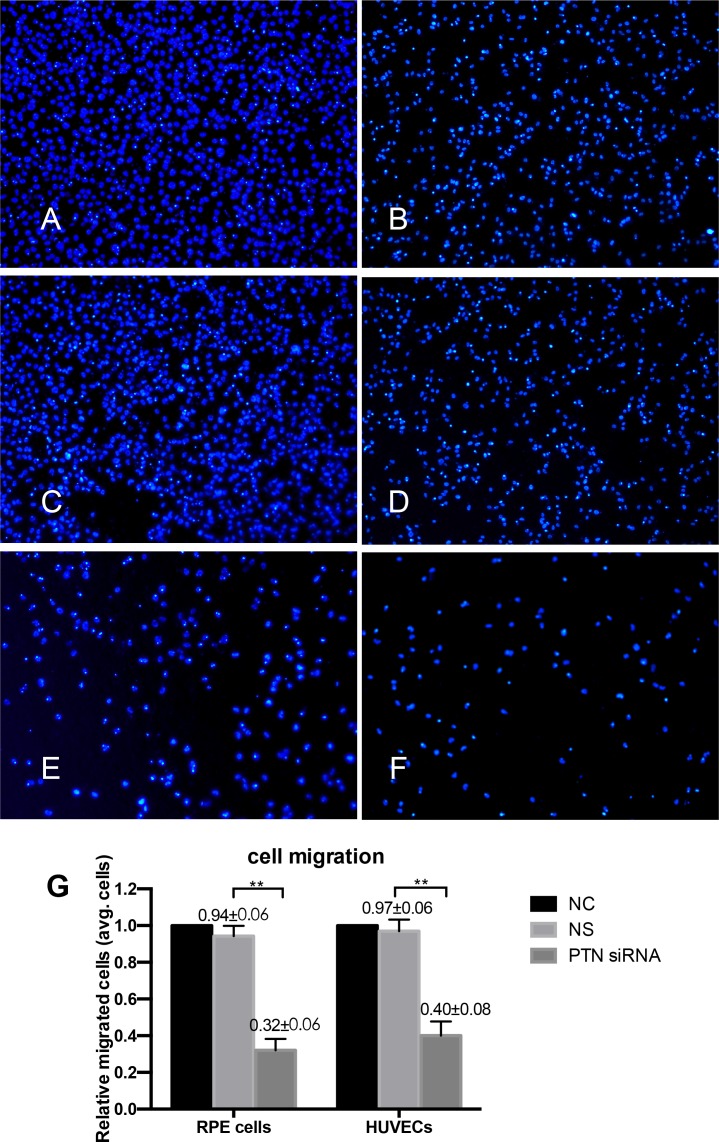
Effect of PTN on cell migration. Cell nuclei were stained with DAPI and are shown as blue dots. Cells from five random view fields were counted, and the average was used for statistical analysis. The migratory activity of PTN-siRNA-treated (E, F) groups was significantly inhibited compared to the NC (A, B) and NS (C, D) groups. Panel G shows the statistical results. The data are presented as the means ± SD. NC was set to 100%. ** P< 0.01.

**Figure 7:**
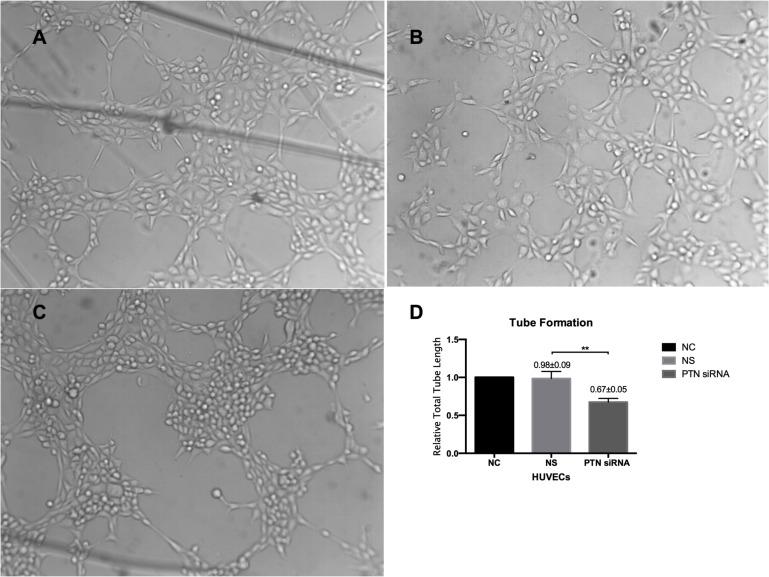
Effects of PTN on HUVEC tube formation. Microphotographs are representative of tube-like structure generation after a 4-h incubation on Matrigel. The length of tube branches per view field was measured. PTN-siRNA-treated HUVECs (panel C) showed an impaired capacity to form a regular vascular network. The length of the angiogenesis network was statistically significantly decreased compared to the control groups. Each experiment was repeated at least three separate times. Data are presented as the means ± SD. NC was set to 100%. **P < 0.01.

A CCK-8 assay was used to evaluate the effects of PTN on cell attachment and proliferation *in vitro*, as stated in the Methods section. In the cell attachment assay, PTN-siRNA-treated cells reduced the adhesive capacities of RPE cells and HUVECs compared with those of the NS-siRNA-treated group (P < 0.01; **Fig. 5A**). Blocking PTN reduced cell proliferation in RPE cells and HUVECs compared with NS cells (P < 0.01; **Fig. 5B**). The role of PTN in the migration of RPE cells and HUVECs was assessed with a Transwell assay. As shown in **Fig. 6**, the mean number of cells that passed through the membrane in the PTN-siRNA-treated groups was significantly lower compared to the control groups (P < 0.01). The Matrigel assay is one of the most widely used methods to evaluate the angiogenesis capacity of endothelial cells *in vitro*. In our study, PTN-siRNA-treated HUVECs exhibited an impaired capacity to form a regular vascular network (**Fig. 7C**). The length of the network was also significantly decreased compared to the control groups (P < 0.01; **Fig. 7D**). There were no differences between the NC and NS groups in any of the cell activity experiments (P > 0.05).

### Cell cycle arrest and apoptosis were induced by PTN depletion

**Figure 8:**
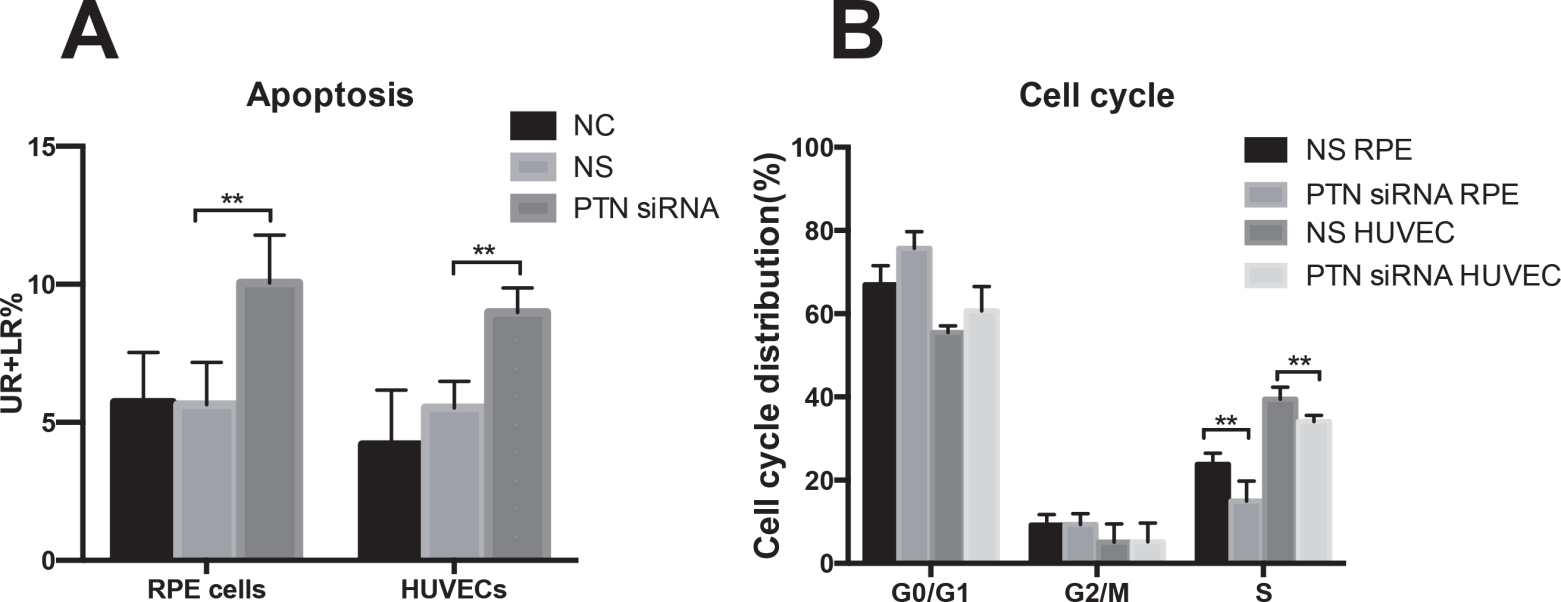
Effects of PTN on cell apoptosis and the cell cycle. (A) The statistical results of PTN on cell apoptosis. The percentage of early apoptotic cells plus late apoptotic cells in the PTN-siRNA-treated group was significantly higher than the controls, indicating that PTN depletion induces cell apoptosis in RPE cells and HUVECs. (B) The statistical data for the cell cycle distributions of NS-siRNA and PTN-siRNA treated groups in RPE cells and HUVECs. PTN-siRNA treatment induces cell cycle arrest in the G0/G1 phase and results in reduced cell numbers in the S phase. Data are presented as the means ± SD and presented in **Table 2**. Each experiment was repeated three times. **P < 0.01.

In our study, the percentage of early apoptotic cells plus late apoptotic cells in the PTN-siRNA-treated group was significantly higher compared to the controls, indicating that depletion of PTN increased cell apoptosis (P < 0.01; **Fig. 8A**). As shown in **Fig. 8B**, depletion of PTN levels in RPE cells and HUVECs induced cell accumulation in the G0/G1 phase of the cell cycle and a marked reduction in the accumulation of cells in S phase compared with the NS group (P < 0.01). There were no differences between the NC and NS groups in the cell cycle and apoptosis (P > 0.05). These results suggested that knockdown of PTN expression induced cellular arrest and apoptosis *in vitro* (**Table 2**).

**Table 2:** Summary of Flow Cytomery data of cell apoptosis and cycle (mean±SD) (P value: NS vs PTN siRNA).

Cell percentage	RPE cells				HUVECs			
	NC	NS	PTN siRNA	P-value	NC	NS	PTN siRNA	P-value
UR+LR%	5.7±1.5	5.6±1.2	10.8±0.8	< 0.01	4.2±1.6	5.52±0.8	9.0±0.7	< 0.01
G2/M+S%	33.5±2.8	33.0±3.8	24.3±3.3	< 0.01	44.2±2.1	44.5±1.3	39.3±4.7	< 0.01

### Phosphorylated ERK 1/2 levels were down-regulated by PTN knockdown

**Figure 9:**
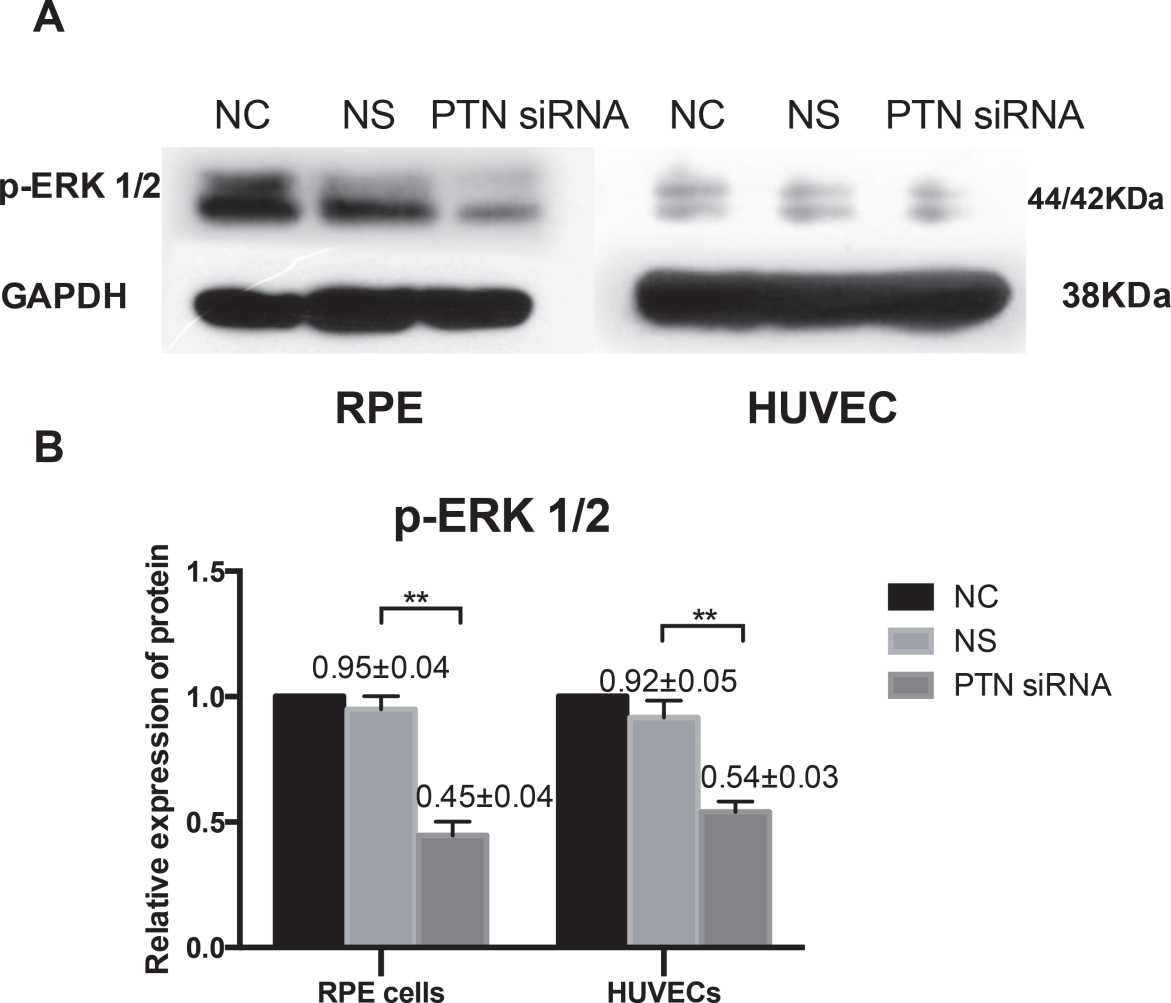
Effects of PTN on ERK 1/2 phosphorylation. Immunoblot imaging (A) and statistical analysis (B) for p-ERK 1/2 signaling pathways. The statistical results show that PTN-siRNA-treated group significantly inhibited p-ERK 1/2 levels. Western blot analyses were repeated three times, and qualitatively similar results were obtained. Data are presented as the means ± SD. **P < 0.01.

The ERK 1/2 signaling pathway plays a key role in DR by mediating VEGF release[41] and regulating cell proliferation[42]. Previous studies[43,44] have reported an increase in ERK signaling in hyperglycemic conditions at the mRNA and protein expression levels. In our study, immunoblot analysis revealed that PTN depletion significantly inhibited the level of p-ERK 1/2 under hyperglycemic and hypoxic culture conditions (P < 0.01, **Fig. 9**).

### Effects of recombinant PTN on VEGF_165_ expression in human RPE cells and HUVECs

**Figure 10:**
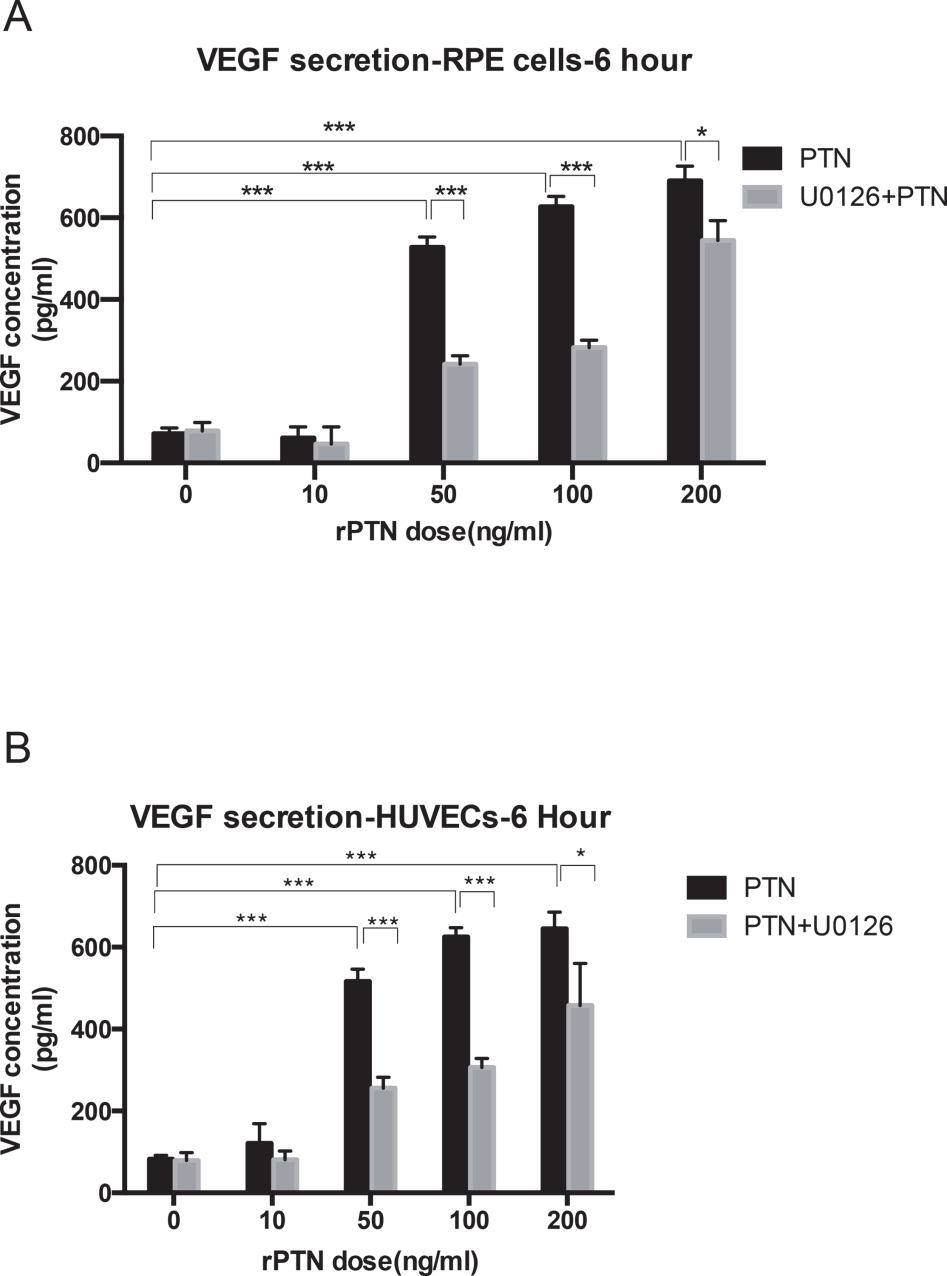
Effects of recombinant PTN (rPTN) on VEGF_165_ expression. The statistical results of secreted VEGF_165_ concentrations, as measured with an ELISA assay at different rPTN concentrations at 6-h incubation timepoints in RPE cells (A) and HUVECs (B). The effects of co-incubation of U0126(10 μM, inhibitor of ERK 1/2) and rPTN on VEGF_165_ expression were also observed. Each experiment was repeated in 6 wells and was duplicated at least three times. Data are presented as the means ± SD and presented in **Table 3**. *P < 0.05 and ***P< 0.001.

To explore the effect of PTN depletion on VEGF expression *in vitro*, we used ELISA to examine the levels of VEGF_165_ secreted into the culture medium. We found that there was no significant difference in the level of VEGF_165_ secretion in the culture medium between the PTN-siRNA–treated cells and the NS cells (P > 0.05; **S3 Fig.**). Then we determined the effect of rPTN on VEGF expression *in vitro*. Our data demonstrated that VEGF expression levels exhibited an ascendant time–response curve after PTN exposure. At 1 and 4 h of incubation with rPTN, the VEGF levels were very low, and no significant differences were observed between groups in RPE cells and HUVECs. At the 6-h time point, there was a striking increase in the secreted VEGF_165_ concentration in all of the rPTN-treated cells under general culture conditions except for the low-dose group (10 ng/ml) (**Fig. 10**, rPTN [50, 100, and 200 ng/ml] groups vs. the 10%FBS group; P < 0.001; [10 ng/ml] group vs. 10%FBS, P > 0.05). At the 24-h time point, the VEGF levels in each group were extremely high compared to the baseline; however, the differences were not significant.

While under the U0126 (10μM) co-incubation condition, secreted VEGF_165_ levels were significantly decreased compared to those in the rPTN-treated groups (**Fig. 10**, rPTN groups vs. U0126+rPTN groups [50 and 100 ng/ml, P < 0.001; 200 ng/ml, P < 0.05]) at the 6- and 24-h time points. The data are presented in **Table 3**. The above results indicated that PTN did affect VEGF_165_ secretion in both RPE cells and HUVECs under general culture conditions and that the ERK 1/2 inhibitor U0126 could inhibit the effects.

**Table 3:** The concentration of VEGF stimulated by rPTN in vitro (mean±SD, pg/ml).

**rPTN concentration (ng/ml)**	**RPE**				**HUVEC**			
	6h		24h		6h		24h	
rPTN	rPTN+U0126	rPTN	rPTN+U0126	rPTN	rPTN+U0126	rPTN	rPTN+U0126
0	71.7±11.2	78.7±16.8	466.4±147.6	269.9±130.6	82.0±8.1	78.7±16.3	793.7±110.1	290.7±88.8
10	61.1±22.3	46.1±34.2	652.7±228.8	539.6±99.3	120.9±39.3	80.7±17.5	738.0±65.7	328.1±31.8
50	527.5±20.5	241.5±17.0	637.2±274.7	310.2±104.6	516.4±24.3	255.8±21.6	797.3±46.4	483.9±151.1
100	627.0±20.5	282.6±14.3	668.7±265.3	455.6±191.3	624.8±18.7	306.4±17.9	939.8±75.5	393.9+159.7
200	690.5±29.1	544.2±39.6	759.5±305.0	356.7±166.1	645.2±33.0	457.5±83.9	1079.2±210.8	450.8±111.7

## Discussion

PTN has been widely studied in angiogenic, fibrotic and neurodegenerative diseases. However, little is known about its effects on diabetic retinopathy, a neurovascular disease. Our results showed that (1) PTN levels in the vitreous body and proliferative membranes were significantly higher in patients with PDR; (2) under hyperglycemic and hypoxic conditions, PTN knockdown inhibited cell attachment, proliferation, migration, and tube formation and promoted cell cycle arrest and apoptosis in RPE cells and HUVECs; (3) PTN knockdown down-regulated ERK 1/2 phosphorylation in RPE cells and HUVECs; and (4) increased VEGF levels were observed in RPE cells and HUVECs stimulated by recombinant PTN, which could be abrogated by the ERK 1/2 inhibitor U0126. Collectively, these findings implied that elevated PTN might participate in the key processes of the development of PDR, likely through ERK 1/2 activation and VEGF regulation. To our knowledge, this study is the first showing the involvement of PTN in PDR.

PTN expression is low or absent in most normal tissues, but in our study, it is markedly elevated in vitreous bodies and fibrovascular membranes from PDR patients compared with the non-diabetic controls. Previous studies revealed that PTN overexpression was detected in many pathologic conditions, including angiogenic, degenerative, inflammatory and neoplastic diseases. Exhibiting a proto-oncogene function, PTN is mainly up-regulated in many types of tumors, such as breast, prostate, and pancreatic cancers[22,23,24], acting as an angiogenic factor. Several studies have demonstrated that PTN enhances the angiogenesis process of tumor cells and that attenuation of PTN expression reduces tumor-induced angiogenesis *in vivo* and *in vitro*[22,28]. In addition, by stimulating the epithelial-to-mesenchymal transition (EMT), PTN affects perineural invasion and metastasis by remodeling the malignant cell microenvironment[25,26,27]. In addition, PTN has been reported to have neovasculogenic effects in ischemic heart myocardial infarction[45] and to potentiate cardiomyocyte apoptosis[46]. It has also been investigated in fibrotic liver tissues, hypertrophic scars[30] and rheumatoid arthritis[31], where it is involved in fibrogenesis and inflammation. Moreover, previous studies have demonstrated that PTN affects cell growth, differentiation, migration, vascularization, and cell apoptosis of various cells to a greater extent than tumor cells, such as endothelial cells, fibroblasts and microglia[40].

In PDR, neovascularization and fibrotic proliferation are predominant features, and the vitreous body is the microenvironment of the retina, precisely reflecting the conditions of the retina. Many growth factors and cytokines have been detected in the vitreous and have been confirmed to take part in the progression of PDR, such as VEGF. In our study, significantly elevated intravitreous PTN concentrations were observed in PDR patients. Considering its angiogenic and proliferative roles in other diseases, we hypothesize that PTN may promote the angiogenesis and proliferation in PDR, thus experiment *in vitro* was conducted subsequently. Additionally, the correlation between PTN and VEGF, the most important growth factor involved in PDR, was also explored in PDR patients. The relationship between PTN and VEGF has been partially demonstrated in other disorders. Kong et al.[47] showed that high PTN expression is accompanied by high VEGF expression in colorectal cancer patients. In our study, all of the patients who received anti-VEGF therapy before surgery that we detected still had high PTN levels, although their VEGF levels were very low. While the sample size is small, at present, our unpublished data on the effect of anti-VEGF therapy on PTN expression in neovascular glaucoma patients complicated by proliferative diabetic retinopathy also have the same results. These findings suggest that PTN levels were not affected by anti-VEGF therapy. Our preliminary result *in vitro* demonstrated that VEGF knockdown did not affect the expression of PTN at mRNA level and in the cell culture supernatant (**S4 Fig.**). Therefore we speculate that PTN may partially mediate angiogenesis independent of VEGF. Considering the patients who are nonsensitive or resistant to anti-VEGF therapy in clinical practice, anti-PTN therapy might be promising. Moreover, PTN is not only detected in the vitreous body of PDR patients but also on their fibrovascular membranes. Fibrovascular membranes are the result of abnormal proliferation in many retinopathies; RPE cells, endothelial cells and glial cells have been reported to play important roles in their formation. As shown in **Fig. 1**, PTN was not only detected in vessels but also in the fibrous-like tissues. We also stained for PTN with the GFAP, a biomarker of glia cells, and found that PTN was also detected around glia cells. The detection of PTN in proliferative membranes from PDR patients instead of EMM patients indicated its possible pathologic role in the formation of PDR membranes. According to our results and other studies, not only endothelial cells could secrete PTN but also RPE cells and glia cells. It has been shown that epiretinal macular membranes include RPE cells and glia cells, but PTN did not be detected in membranes from EMM patients. Combining our results that PTN knockdown *in vitro* could inhibit cell attachment, proliferation and migration, which were the key processes of proliferative membrane formation, we think PTN might involve in the development of membranes in PDR. A limitation is that PTN receptors have not been explored in proliferative membranes. Clinically, our findings highly suggest that PTN may play an important role in the progression of PDR.

We then explored the effect of PTN *in vitro*. In PDR, vascular endothelial cell abnormality is mainly responsible for the neovascularization and dysfunction of the RPE, such as its transition to a proliferative and migratory phenotype, is responsible for proliferative vitreoretinopathy and indirectly influences neovascularization by secreting growth factors, while hyperglycemia and hypoxia are considered to be key factors responsible for cell dysfunction. Thus, to observe the effects of PTN *in vitro*, we incubated RPE cells and HUVECs under hyperglycemic and hypoxic conditions to simulate the *milieu interne* of PDR patients. In our study, RPE cells and HUVECs both expressed PTN, and there was no transfection-induced cytotoxicity apparent in the cells. The effectiveness of PTN-siRNA transfection was then determined, and subsequent results demonstrated that PTN downregulation inhibited cell attachment, proliferation, migration, and tube formation and increased cell cycle arrest and apoptosis in RPE cells and HUVECs, which means that PTN knockdown could inhibit the key processes of proliferative membrane formation and neovascularization in PDR. These observations were similar to previously published reports in other fields[36]. Therefore, it is reasonable to predict that PTN depletion therapy holds promise for the treatment of PDR. However, more *in vivo* evidence is needed, which our laboratory is currently developing.

Furthermore, we investigated the possible signaling pathway of PTN *in vitro*. The primary receptor for PTN is receptor protein tyrosine phosphatase (RPTP) β/ζ. Through inactivation of its receptor, PTN signaling leads to increased tyrosine phosphorylation of different substrate proteins of RPTPβ/ζ, including β-catenin, β-adducin, Fyn, GIT1/Cat-1, and P190RhoGAP[48]. Recently, PTN was found to activate anaplastic lymphoma kinase (ALK) through the PTN/RPTPβ/ζ signaling pathway[48]; and the rescue of fibroblast apoptosis by PTN is mediated through ALK receptor–dependent AKT and ERK activation[49]. The ERK 1/2 signaling pathway plays a key role in DR and previous studies[43,44] have reported an increase in ERK signaling in hyperglycemic conditions at the mRNA and protein expression levels. In our study, phosphorylated ERK 1/2 levels were significantly decreased in PTN-siRNA-treated cells compared to the control groups, indicating that ERK 1/2 might be a downstream signal protein of PTN and mediate cell function, which is consistent with previously published studies[40,50]. However, the underlying mechanism of ERK activation by PTN is still not fully understand, we have detected the expression of RPTPβ/ζ not ALK in HUVECs and RPE cells by RT-PCR. A study revealed that in osteoblasts in which no reports show ALK expression, the EGFR trans-activation was reported to be the key mediator for PTN-induced AKT/ERK activation[50]. In another study, RPTPβ/ζ was demonstrated to transduce the PTN signal to regulate cell migration and tube formation under the interplay between αvβ3 integrin and nucleolin[51]. Therefore, we speculate that PTN may bind its receptor RPTPβ/ζ and then activates ERK 1/2, but the underlying mechanism needs more exploration.

Additionally, the correlation between PTN and VEGF was explored *in vitro*. First, the effect of PTN depletion on VEGF expression was evaluated. In the cell supernatants, there were no significant differences in VEGF levels between PTN-siRNA-treated cells and the NS-siRNA-treated cells, likely because the basal secretion in cells was low, and the VEGF secretion is regulated by many other signaling pathways. We then determined that recombinant PTN could increase VEGF levels in the supernatants of RPE cells and HUVECs in an ascendant time–response manner, which could be the result of either the upregulation of VEGF expression or the downregulation of utilization. Corroborating our results, a study by Liu et al.[52] showed that PTN increased the mRNA and protein expression of VEGF in human peritoneal mesothelial cells, and the effect was decreased in the presence of the blocking peptide of PTN. In our study, significantly decreased VEGF expression after co-incubation of rPTN with the ERK 1/2 inhibitor U0126 was observed. This finding suggested that the ERK 1/2 pathway may participate in regulating VEGF secretion stimulated by PTN *in vitro*. According to the results above, it is reasonable to speculate that PTN has a relationship with VEGF. At present, there is no sufficient evidence to prove that PTN totally acts as an angiogenic factor independent of VEGF, although our clinical data suggested that PTN expression was not affected by anti-VEGF therapy and VEGF expression was not affected by PTN depletion *in vitro*. Taken together, our laboratory results suggest that PTN may partially mediate angiogenesis and proliferation through ERK 1/2 activation and VEGF regulation.

In summary, our results implied that elevated PTN in PDR patients might participate in the critical processes of the development of PDR, most likely playing roles in angiogenesis and proliferation, possibly by activating the ERK 1/2 pathway and regulating VEGF secretion. These findings suggest that PTN may become a new target for therapeutic intervention in PDR.

## Supporting Information

S1 Fig Detection of PTN and GFAP on epiretinal membranes from PDR patients. Micrographs show immunofluorescence double staining of epiretinal membrane sections from PDR patients for PTN (A) and GFAP (B). Nuclei were stained with DAPI (C). Image in the panel D is merged and panel E is the enlargement of the box in panel D. Double staining revealed expression of PTN and GFAP in PDR membranes. PTN expression was detected in GFAP-labeled cells (panel E, short arrow). The expression of PTN was also detected in other field (panel D, long arrow). Bar, 75 μm.
(TIF)

S2 Fig Cell cytotoxicity induced by transfection. Cells were transiently transfected with transfection reagent (HiPerfect [HF]; Qiagen) and control siRNA (NS) and incubated for 48 h. The effect of transfection reagent and NS siRNA on apoptosis of human RPE cells and HUVECs was present in panel A. The normal living cells (bottom left quadrants) showed low Annexin V and propidium iodide staining. The early apoptotic cells (bottom right quadrants) showed high Annexin V staining but low propidium iodide staining. The late apoptotic cells (top right quadrants) showed intense Annexin V and propidium iodide staining. The percentages of cells in the quadrants are indicated within the quadrant. Representative results of three separate experiments are shown. The extent of inhibition of cellular viability was measured by the CCK-8 assay (panel B). Data are the mean ± SD of results from at least three independent experiments.
(TIF)

S3 Fig Effects of PTN depletion on VEGF secretion in RPE cells. After transfection, the culture medium was harvested. VEGF released into the culture supernatant was measured by ELISA. There was no significant difference in the level of VEGF secretion in the culture medium between the NS siRNA group and PTN siRNA group (P > 0.05), while the levels of VEGF in PTN-siRNA-treated cells were lower than the control group. Data are the mean ± SD of results from three independent experiments. The Normal group was set to 100%.
(TIF)

S4 Fig Effect of VEGF depletion on PTN expression in vitro. Knockdown of VEGF was achieved via small interference (si)RNA in human RPE cells and HUVECs. VEGF expression was significantly knocked down in VEGF-siRNA treated groups as measured by real-time PCR (A). After siRNA transfection for 48h, the culture medium was harvested and total RNA of cells was isolated. The expression of PTN at mRNA level (B) in human RPE cells and HUVECs was detected by real-time PCR. There was no significant difference between the NS siRNA group and VEGF siRNA group (P > 0.05). PTN released into the culture supernatant was measured by ELISA (C). There was no significant difference in the level of PTN secretion in the culture medium between the NS siRNA group and VEGF siRNA group (P > 0.05). The NC was set to 100%. Data are the mean ± SD of results from three independent experiments.
(TIF)
