# Supplementary figures and images for: The Effects of Pleiotrophin in Proliferative Diabetic Retinopathy
Source: PLoS One. 2015 Jan 24;10(1):e0115523. doi: 10.1371/journal.pone.0115523 (PMC4305314; doi:10.1371/journal.pone.0115523)

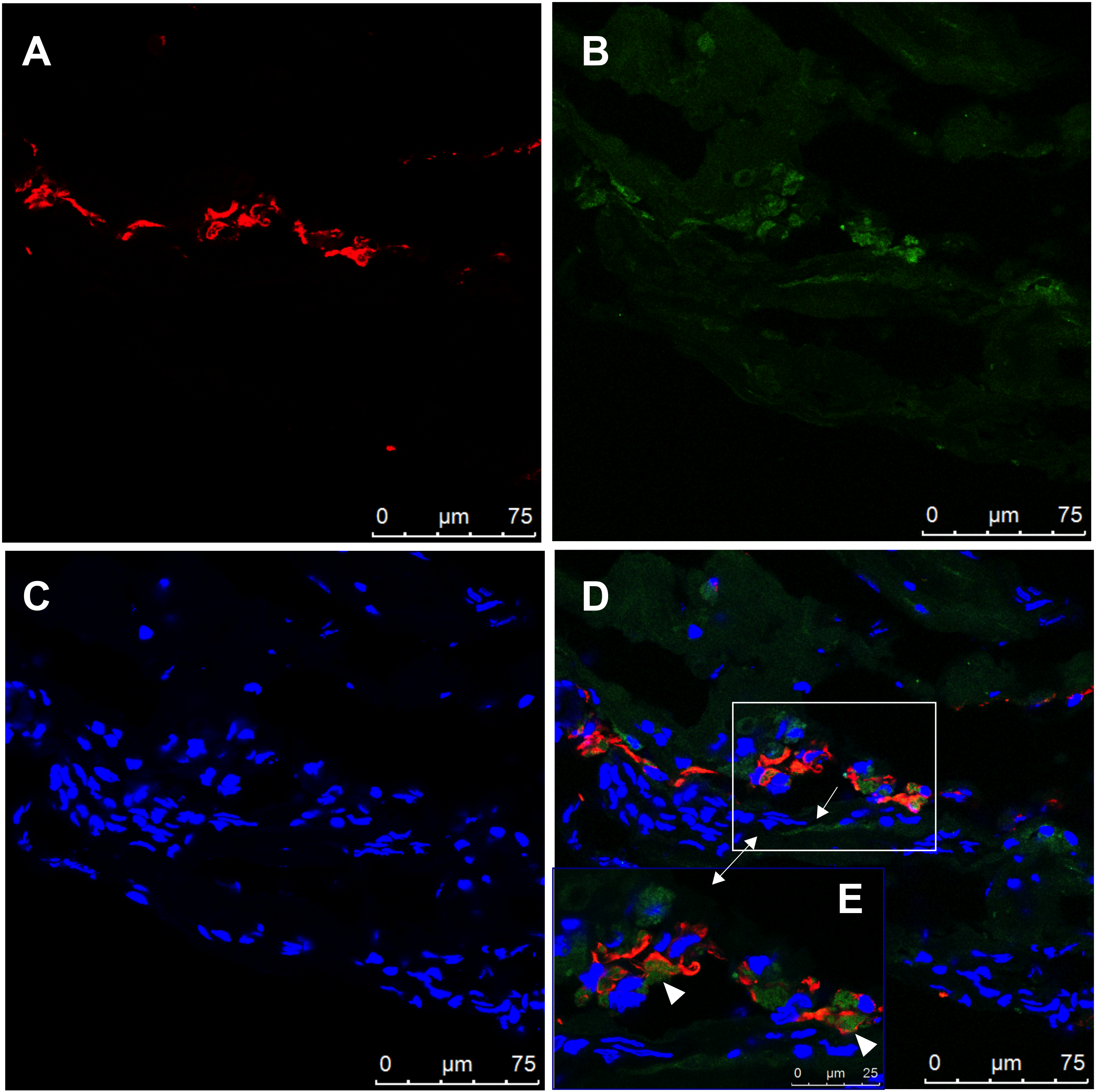

Supplement: S1 Fig — Micrographs show immunofluorescence double staining of epiretinal membrane sections from PDR patients for PTN (A) and GFAP (B). Nuclei were stained with DAPI (C). Image in the panel D is merged and panel E is the enlargement of the box in panel D. Double staining revealed expression of PTN and GFAP in PDR membranes. PTN expression was detected in GFAP-labeled cells (panel E, short arrow). The expression of PTN was also detected in other field (panel D, long arrow). Bar, 75 μm. (TIF) [file pone.0115523.s001.tif]

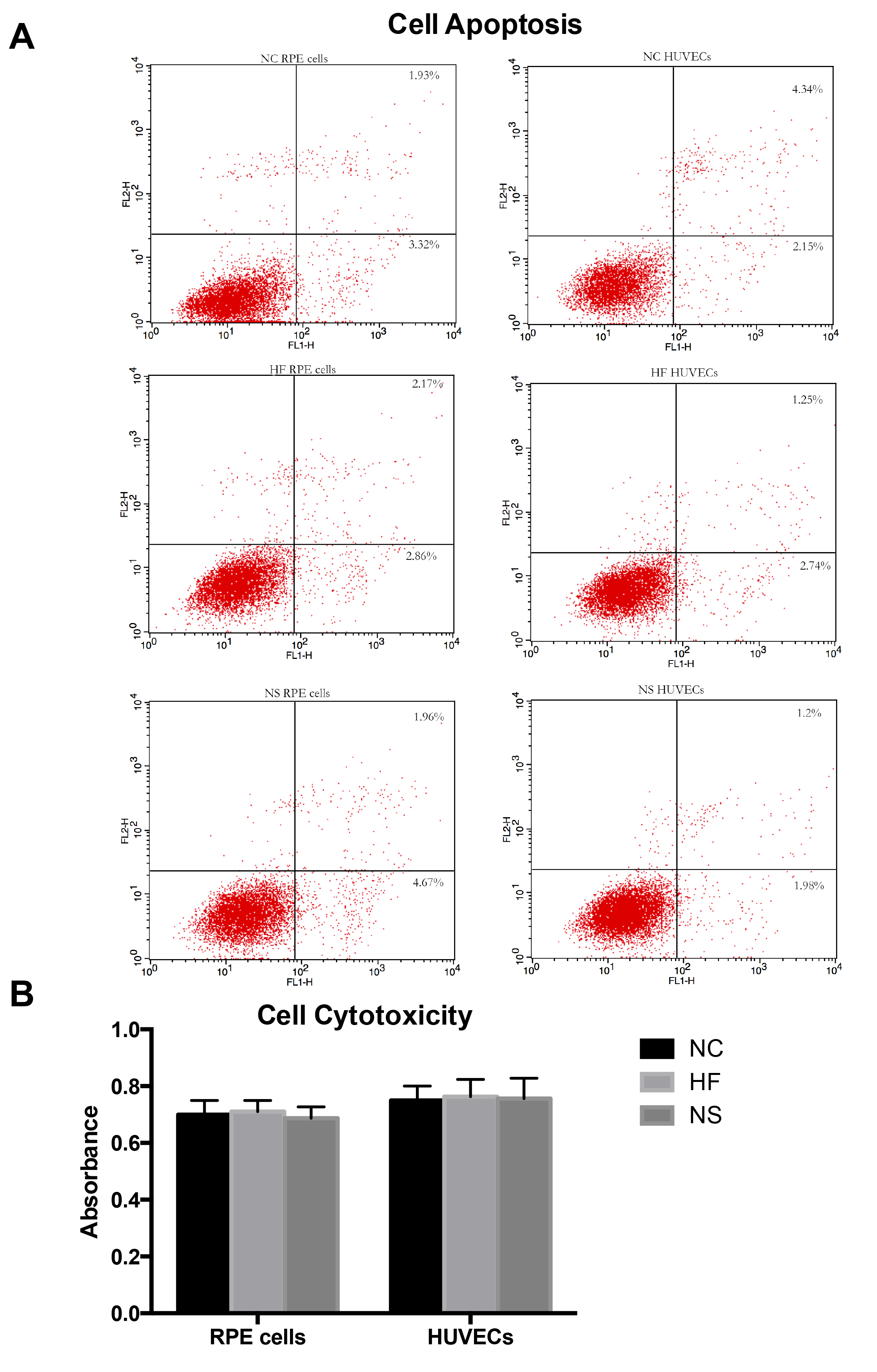

Supplement: S2 Fig — Cells were transiently transfected with transfection reagent (HiPerfect [HF]; Qiagen) and control siRNA (NS) and incubated for 48 h. The effect of transfection reagent and NS siRNA on apoptosis of human RPE cells and HUVECs was present in panel A. The normal living cells (bottom left quadrants) showed low Annexin V and propidium iodide staining. The early apoptotic cells (bottom right quadrants) showed high Annexin V staining but low propidium iodide staining. The late apoptotic cells (top right quadrants) showed intense Annexin V and propidium iodide staining. The percentages of cells in the quadrants are indicated within the quadrant. Representative results of three separate experiments are shown. The extent of inhibition of cellular viability was measured by the CCK-8 assay (panel B). Data are the mean ± SD of results from at least three independent experiments. (TIF) [file pone.0115523.s002.tif]

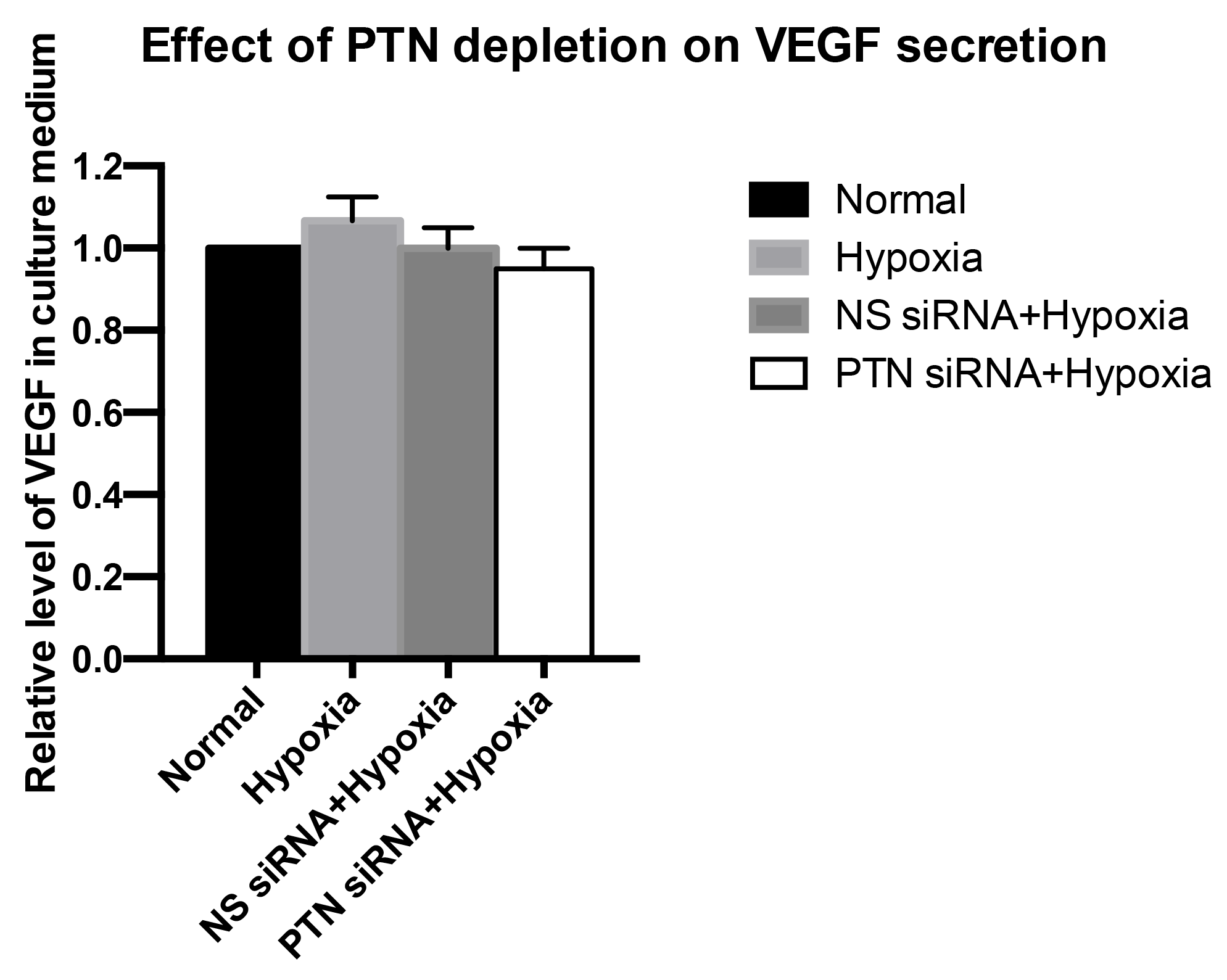

Supplement: S3 Fig — After transfection, the culture medium was harvested. VEGF released into the culture supernatant was measured by ELISA. There was no significant difference in the level of VEGF secretion in the culture medium between the NS siRNA group and PTN siRNA group (P > 0.05), while the levels of VEGF in PTN-siRNA-treated cells were lower than the control group. Data are the mean ± SD of results from three independent experiments. The Normal group was set to 100%. (TIF) [file pone.0115523.s003.tif]

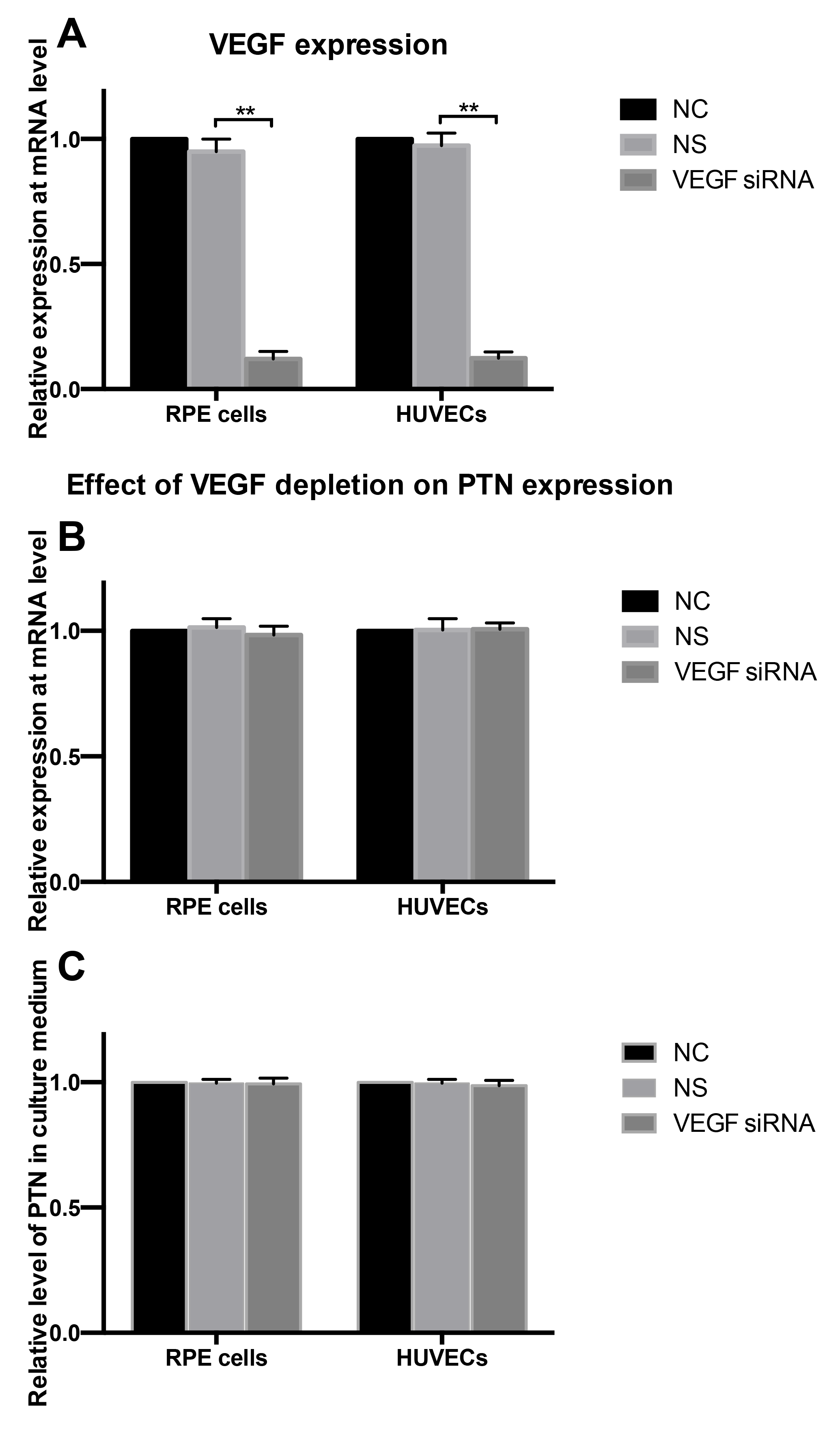

Supplement: S4 Fig — Knockdown of VEGF was achieved via small interference (si)RNA in human RPE cells and HUVECs. VEGF expression was significantly knocked down in VEGF-siRNA treated groups as measured by real-time PCR (A). After siRNA transfection for 48h, the culture medium was harvested and total RNA of cells was isolated. The expression of PTN at mRNA level (B) in human RPE cells and HUVECs was detected by real-time PCR. There was no significant difference between the NS siRNA group and VEGF siRNA group (P > 0.05). PTN released into the culture supernatant was measured by ELISA (C). There was no significant difference in the level of PTN secretion in the culture medium between the NS siRNA group and VEGF siRNA group (P > 0.05). The NC was set to 100%. Data are the mean ± SD of results from three independent experiments. (TIF) [file pone.0115523.s004.tif]
